# Everyday hazards and vulnerabilities amongst backyard dwellers: A case study of Vredendal North, Matzikama Municipality, South Africa

**DOI:** 10.4102/jamba.v7i1.210

**Published:** 2015-11-30

**Authors:** Patricia J. Zweig

**Affiliations:** 1 Department of Geography & Environmental Studies, Stellenbosch University, South Africa

## Abstract

The populations of many small towns in South Africa continue to expand unmatched by parallel economic growth, entrenching high levels of poverty. The town of Vredendal, located close to the national route between Namibia and Cape Town in South Africa, is a West Coast development node and an emergent industrial and processing area that continues to attract an influx of people seeking economic opportunities. This is challenging the capacity of the local municipality, which has a waiting list for state-provided low-cost housing units, whilst the provision of adequate infrastructure to meet growing local need is also a developmental concern. In the suburb of Vredendal North this has resulted in the proliferation of unplanned informal dwellings in the backyards of formalised low-cost housing areas. Largely overlooked by urban researchers, little is known or understood about small town backyard populations. This prompted a brief study of Vredendal North backyard dwellers commissioned by the local municipality to identify their everyday hazards and livelihood vulnerabilities to inform future development planning. A community workshop identified critical development needs and suggested that backyard dwellers in small towns experience similar living conditions and hazards to those in the cities, although underlain by some unique differences.

## Introduction

A growing body of global (Cohen 2004), regional (Holloway *et al.* 2013) and South African academic literature (Hoogendoorn & Nel 2012; Nel & Hill 2008) suggests that the informal and low-income populations of many small towns are growing at an unsustainable rate, unmatched by parallel economic growth and entrenching high levels of poverty. In South Africa, this situation is being further exacerbated by the arrival of migrating job seekers, amongst them many displaced farm workers and their families as well as increasing numbers of foreign migrants. Although in terms of regional spatial development planning, many small towns in South Africa have been recognised as critical development nodes (Atkinson 2008; Van der Merwe *et al.* 2004), local authorities have generally received modest state support for the provision of the resources and capacities required for them to take advantage of their pivotal positions (Nel 2001; Nel & Rogerson 2007; Wienecke 2012).

Typical of such small towns, Vredendal, located on the national route between Namibia and Cape Town, although principally an agricultural area, is also a West Coast development node. As such it is an emergent industrial and processing area that is attracting an influx of people seeking economic opportunities. It is also experiencing on-going in-migration from outlying and increasingly unsustainable arid peripheral areas.

Vredendal North, the study area, is a low-income suburb situated on the outskirts of Vredendal where levels of unemployment are critically high. With an estimated housing backlog of some 3500 housing units (Matzikama District Municipality 2013), it is common for existing formal houses to accommodate multigenerational households consisting of two or even three families, with extended family or tenants often living in informally-constructed backyard dwellings.

The proliferation of backyard dwellings is contributing to an increasingly risk-prone environment, posing a critical development challenge in terms of infrastructural need and service provision that a small town such as Vredendal, given its small tax base, is challenged to afford (Paquet & Donaldson 2011). Whilst the Integrated Development Plan of the Matzikama local authority acknowledges these challenges (Matzikama District Municipality 2013), a recent survey of small towns in the Western Cape (Van Niekerk *et al.* 2010) estimates that Vredendal has a medium-level potential in terms of its economic, natural, infrastructural and institutional capacity. The optimisation of future development potential, however, requires an informed understanding of local development issues. In this regard the living conditions and the associated risk profile of the growing number of backyard dwellers are poorly understood by decision-makers.

Although living in seemingly more formalised environments, South African studies have repeatedly shown that backyard dwellers are generally marginalised and vulnerable, surviving in suboptimal living conditions with constrained access to resources and often exploited by their landlords (Bank 2007; Carey 2009; Crankshaw, Gilbert & Morris 2000; Gunter 2014; Lemanski 2009; Morange 2002; Shapurjee & Charlton 2013). Backyard accommodation is often viewed as a temporary measure, offering a flexible housing option to serve short-term employment opportunities, better access to services, reduced commuting costs whilst also providing large numbers of single men, female-headed households and foreign migrants an affordable housing option. However, to date most studies have been metropolitan or city-based in context, with little attention paid to backyard dwellers in nonmetropolitan areas where local populations are also growing unsustainably. Indeed, Donaldson and Marais (2012) and Visser (2013) have highlighted a definite bias amongst urban researchers towards metropolitan or city-based research.

Drawing on South African census data, a recent report (Human Sciences Research Council 2013) suggests that backyard dwellings have far less importance outside of the city. Contesting this generalisation, this study suggests that small towns outside of the city may reflect a somewhat different reality. The research, which predated the 2011 national census, investigated the considered importance of backyard dwelling as an alternative housing option in Vredendal North, exploring the particular vulnerabilities and hazardscapes manifested amongst local backyard dwellers. The research, though brief, revealed that although backyarders in this nonmetropolitan context experience similar everyday hazards and vulnerabilities to city-dwellers, they manifest some unique differences in terms of fundamental risk drivers and the pervasive and cumulative effects of these on the larger community.

## Research objectives

Working in collaboration with the Matzikama local authority, which was concerned to understand the nature of the living conditions of backyard dwellers in Vredendal North, a community risk and vulnerability assessment was undertaken to provide the municipality with empirical information. The project was conceived as a service learning project for Honours students at the University of Stellenbosch as part of a Disaster Risk Studies module and was carried out in March 2012. The results were provided in a formal report to the municipality and to the community of Vredendal North to inform integrated development planning and risk reduction initiatives.

## Project methodology

Adopting a participatory research approach involving members of the Vredendal North backyard dweller community, a community risk and vulnerability assessment was undertaken over several days. As Wisner (2010:135) suggests, employing such methods causes people ‘to become more conscious of their situation and their own knowledge and practice’. In adopting this methodology the research aimed not only to collect information on behalf of the local authority but also to involve the backyard dwellers themselves in an assessment of their own living environment.

The project was conducted prior to the 2011 census when the municipality did not yet know the extent of backyard dwellings in Vredendal North, why they were growing in number, the nature of living conditions and the types of hazards prevalent amongst such dwellings. The project, in attempting to fill this gap in municipal knowledge, could not systematically survey the whole suburb due to time constraints, but instead tasked groups of students to visit a geographically representative sample of backyard homes in a designated area. Essentially an exercise in participatory research techniques and interview skills, the project tasked each group to identify the extent of dwellings in their sample area, describe the nature of living conditions and identify prevalent hazards amongst these dwellings. Based on their community survey and the collaborative community workshop that they facilitated, each student group had to map the extent of backyard dwellings in their survey area, describe the conditions and the hazard profile and, after drawing some conclusions, make recommendations for integrated development planning and risk reduction.

Prior to the commencement of fieldwork the students held a discussion session with key municipal stakeholders during which historic and prevailing developmental issues in Vredendal North were debated and discussed. The perspectives of the local authority provided critical initial insights ahead of the fieldwork. This was followed by a transect walk, accompanied by local community development workers, to meet with backyard dwellers and their landlords and observe first-hand the living conditions amongst backyard dwellings. The following day the municipality assisted the students by publicly inviting backyard dwellers who had not been approached during the survey to a community workshop using a loud hailer.

During the workshop backyard dwellers working with students trained in the use of participatory research tools interrogated the nature of hazards and everyday risks in the backyard dwellings of Vredendal North. They also explored the nature of locally manifested development issues impacting on their livelihoods and shaping local vulnerabilities. The tools, which have been employed in a variety of development contexts for several decades across many disciplines, are based on adult educational principles historically promoted by Frere (1970), later employed by Chambers (1994), but more recently adapted by Mercer *et al.* (2008) and Holloway and Roomaney (2008). They are appropriate for research amongst less-educated adult community members, enhancing their engagement.

In recognising the value of collective insights regarding local issues, the highly visual nature of the tools employed, provokes conversation and discussion, stimulating reflection and debate amongst community members. They also engender self-confidence amongst participating community members, many of whom may be encouraged to contribute their own perspectives for the first time. The tools included hazard identification and prioritisation exercises, flow diagrams to determine the causes and effects of identified hazards, seasonal calendars to identify patterns and trends in vulnerability, Venn diagrams to interrogate the nature of relationships and available social capital, community risk mapping, and risk history tables.

## Backyard dwellers

### Contextual framing within the South African housing debate

It is widely acknowledged (Baumann 2003; Lemanski 2009; Napier 2007) that from the late 1960s the South African apartheid government’s moratorium on the provision of housing for a growing urban black population in terms of the state’s separate development policies created a legacy that underlies the critical and growing shortage of accommodation manifest in urban areas today. This had the effect of encouraging informal urban tenancy, in rooms within existing houses (Crankshaw & Parnell 1996; Lemanski 2011), in shared hostel accommodation (Ramphele 1993) and later in backyard shacks which proliferated with the rescinding of apartheid legislation from the late 1980s onwards (Gilbert *et al*. 1997; Lemanski 2009).

Subsequently backyard dwellings have become a ubiquitous element of the South African urban landscape. The first post-apartheid census in 1996 recorded more than 400 000 people living in informal backyard dwellings (Morange 2002). Indeed, this was the first census to differentiate between informal dwellers living in backyards and those in informal settlements (Lemanski 2009). A later survey (South African Institute of Race Relations 2008) found that this number had grown to over 590 000 households, constituting approximately a third of all informal households in South Africa with the remainder residing in informal settlements (Lemanski 2009). Contrary to prevailing perceptions about the untamed growth of informal settlements, the proportion of households living in backyard dwellings had actually been increasing more rapidly (Lemanski 2009). A comparison of census data in 2001 and 2011 (see Table 1) illustrates this continuing reality as state housing delivery programmes fail to keep pace with continued demand (Statistics South Africa 2012). It is interesting to note that whilst the last decade has seen the number of backyard dwellers increase only fractionally nationally, the Western Cape Province, where the study was conducted, has experienced a significant increase in backyard dwellings over the same period – particularly informal structures (see Table 1).

**TABLE 1:** Changes in the number of backyard dwellers between 2001 and 2011.

Census	Total number houses	House or flat or room in backyard	Informal dwelling or shack in backyard	All backyard dwellings	Backyard dwellings as % of total
**Census 2001**					
South Africa	11 778 121	411 960	460 027	871 987	7.40%
Western Cape	1 210 926	24 922	46 957	71 879	5.94%
Western Cape housing as percentage of South Africa	10.28%	-	Western Cape backyards as percentage of South Africa	8.24%	-
**Census 2011**					
South Africa	14 450 162	422 828	712 199	1 135 027	7.85%
Western Cape	1 634 001	23 887	105 280	129 167	7.90%
Western Cape housing as percentage of South Africa	11.31%	-	Western Cape backyards as percentage of South Africa	11.38%	-

The significance of backyard accommodation has been generally disregarded by state housing policies that have focused instead on the large scale delivery of low-cost housing, (Carey 2009; Crankshaw *et al.* 2000; Dewar 1997; Huchzermeyer 2001; Watson 2009; Watson & McCarthy 1998) although they have continued to grow steadily in number (Beal, Crankshaw & Parnell 2002). State housing programmes have unintentionally also provided opportunities for the growth of backyard dwellings in new housing areas (Lemanski 2009; Shapurjee & Charlton 2013), catering for immediate or extended family members and rent-paying tenants (Bank 2007; Crankshaw *et al*. 2000; Lemanski 2009; Morange 2002). Although as Bank (2007:20) suggests ‘most municipalities have not extended basic services to backyard residents’, they do generally acknowledge that backyard dwellings constitute an important alternative source of accommodation in light of municipal housing shortfalls and long waiting lists (Paquet & Donaldson 2011). However, backyard accommodation is still generally viewed as a transitional step to the provision of formalised housing (Bank 2007).

Globally the renting of informal forms of accommodation to tenants has become an effective livelihood strategy employed by the urban poor, answering to the need for affordable housing (Carey 2009; Gilbert 2003; Lemer 1987; Rakodi 1995). It has been estimated, for example, that in Africa and Asia informal rental provides over half the housing needs of the population in urban areas, and a third of those in Latin America (Gilbert *et al*. 1997). However, a comparative study conducted in Brazil and South Africa revealed that whilst informal rental has been a livelihood strategy in Brazil for some time, it was only regularly employed to supplement household income by poor South African households more recently (Gilbert *et al.* 1997) and even today is generally a subsistence strategy for the landlord rather than a profit-making endeavour (Shapurjee & Charlton 2013). Another significant difference is that in South Africa the tenant rather than the landlord generally constructs the dwelling (Gilbert *et al.* 1997), serving to maintain rents at affordable levels. Despite research-based evidence substantiating the importance of backyard dwellings as an affordable housing option in South Africa, and although the need for a regulatory framework has been identified to improve the living conditions and safeguard the rights of both backyard dwellers and landlords (Carey 2009; Charlton, Silverman & Berrisford 2003), no fundamental policy shift has occurred.

Thus, the Matzikama Municipality’s concern to identify and understand the nature of the vulnerabilities and hazards prevalent amongst the backyard population of Vredendal North for longer-term planning purposes is an exciting departure from Bank’s (2007:20) suggestion that municipalities are generally indifferent to the plight of backyard dwellers, refusing to ‘acknowledge their long existence’.

## Backyard dwelling in Vredendal North

### What are the realities?

Vredendal North backyard dwellers appear to face the same risks commonly found in low-income areas throughout South Africa, namely, poorly constructed and fragile housing, poor sanitation and unhygienic living environments, constrained access to water and ablution facilities, limited access to electricity, fire hazards, high levels of crime and endemic substance abuse. These risks are rooted in and related to several key underlying developmental factors such as shrinking employment opportunities and limited household income.

Houses in Vredendal North today commonly accommodate one or more backyard dwellings, although the distribution of such dwellings varies spatially, with few backyards found in the higher income area on the eastern side of the suburb. Generally backyard dwellings accommodate three to four people, but overcrowding is common, with five or more people often found sharing one small dwelling. Most dwellings consist of a single room used for all daily tasks. However, items of furniture are often creatively used to divide different living areas. Whilst roughly a quarter of dwellings surveyed have an additional room equipped as a kitchen, most do not and many use the kitchen of the landlord.

**FIGURE 1:**
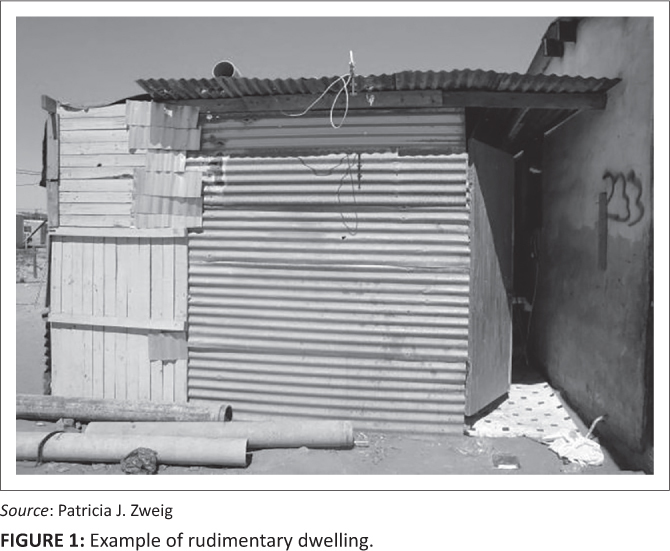
Example of rudimentary dwelling.

**FIGURE 2:**
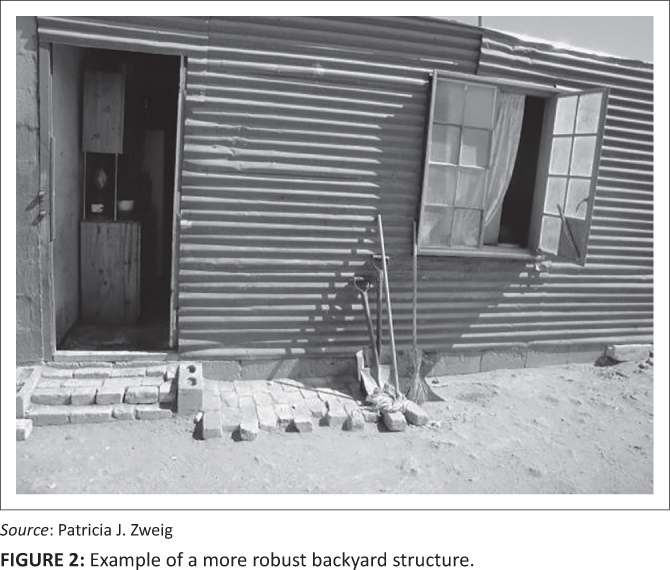
Example of a more robust backyard structure.

Although commonly constructed from recycled materials such as pieces of wood, corrugated zinc sheeting and other building materials, many backyard dwellings in Vredendal North are built more robustly. Generally the dwelling tends to be more solidly and durably constructed, often even built with brick and mortar or purchased as a prefabricated Wendy house^1^, when the backyard dweller is closely related to those living in the main house. The appearance of the dwelling is, thus, often a key indicator of the nature of the relationship between the tenant and the landlord, with crudely constructed structures generally belonging to those with no kinship ties to the landlord. Such ‘shacks’ are generally built by tenants and not by the landlord. Thus, where no kinship exists between the landlord and the tenant there is less security of tenure and therefore limited investment in the construction or repair of the backyard dwelling. Figures 1 and 2 demonstrate this observable difference in housing quality across the landscape. The transect walk, which incorporated a visit to 60 backyard households spread across the Vredendal North community, found higher levels of observable risk amongst casual and unrelated tenants.

According to Morange (2002), tenants prefer to rent in an area that they know well or originate from, are generally well-integrated into the area, building strong neighbourhood relationships, with critical mutual support mechanisms developing between tenants and landlords. Reflecting this trend, most Vredendal North backyard dwellers were found to have been born and raised in the area and generally related to their landlord. Roughly a quarter of Vredendal North backyard dwellers are thus not tenants in the real sense of the word, but older or married children living with their parents, or other family members. This has changed little since as an earlier study (Vorster, Muller & Roussouw 1997), which established that 58.3% of households in Vredendal North were accommodating two generations, with over a third comprising three and even four. Some backyards accommodate kin who have migrated from arid outlying villages and settlements. Kinship links facilitate access to local rental options that are denied to those considered to be ‘outsiders’ or strangers.

Generally backyard households survive on a low average household income, supported by a single bread-winner. Although more male-headed backyard households were found, slightly less than half were female-headed. This differs significantly from a metropolitan survey that showed a much higher frequency of female-headed backyard households (Carey 2009). The age of household heads in Vredendal North varies significantly, from those in their early twenties to those of advanced years, clearly indicating that backyard dwellings accommodate every age group, from single young men to young couples, families with children to pensioners. However, very few backyard dwellings were found to house single people, once again differing markedly from Carey’s (2009) survey in major South African cities, for example, that found the majority of tenants to be single young men. Periods of tenancy also vary from periods spanning ten to fifteen years to shorter tenancies of only a few years or even several months, reflecting two local realities: the long term tenancy of local residents with no other housing options and the expansion of backyard stock to accommodate growing numbers of new home seekers.

In Vredendal North, however, the decision to rent a backyard dwelling is not only governed by the lack of alternative housing options, but is also driven by economic necessity. High levels of unemployment and low household incomes make backyard rental the only affordable option for many, whilst local landlords will often negotiate late or staggered payments to assist struggling tenants. The study revealed that much local employment is only part-time, contract-based or seasonal in nature. With limited skills most local residents must accept unskilled and poorly paid work, such as farm labour, gardening or domestic cleaning, forcing high levels of grant dependency. Thus, household incomes are often determined by the time of the year, with winter being a time of constrained income and particular hardship.

The research also probed why backyarders had not chosen to live in the nearby informal areas, where living conditions are similarly rudimentary, generally less dense, and provided with free electricity and water supply. The prevailing view was that the informal areas are dangerous and crime-ridden places, with most criminals emanating from such areas. Morange’s (2002:11) study in Port Elizabeth similarly found that one of the perceived benefits of living in a backyard is that it offers a safer living environment whilst ‘informal settlements are perceived as changing, unstable and frightening spaces’. Studies by Carey (2009) and Shapurjee and Charlton (2013) reinforce this concern for security, suggesting that the presence of people living in the backyard significantly reduces the risk of crime for both tenant and landlord, offering opportunities for increased vigilance and shared responsibility.

Whilst living in an informal settlement once served to reduce the threat of eviction by hostile landlords (Lemanski 2009), Shapurjee and Charlton (2013:658) suggest that times have changed so that many tenant–landlord relationships today manifest a kind of ‘codependency’ characterised by increasing reciprocity. Lemanski (2009:475) suggests that ‘poor tenants are more likely to rent from poor landlords’ with the circumstances of the landlord often little better than that of the tenant. Indeed, during the transect walk the homes of many landlords were frequently found to be similarly dilapidated and crowded. Despite this, some backyard dwellers insisted that they felt excluded from the community because of what they perceived to be their ‘low social status’ that sets them apart from those who live in formal housing (field notes).

Due to the use of building materials such as wood and tin, most backyard dwellings in Vredendal North are poorly insulated and subject to extreme internal temperatures, suffering intense heat during the summer months (often over 30 °C), whilst the area is prone to fog throughout the year, becoming very cold during winter. Evidence of mould and damp was observed inside backyard dwellings throughout the study area. Such conditions are known to exacerbate the living conditions of those suffering from *Tuberculosis* (Darbyshire 1995), a high local incidence of which was reported by the local clinic although they were not permitted to provide statistical evidence of this.

Access to water supply and sanitation is generally controlled by landlords, with few backyard dwellings provided with an independent water supply and most found to have no ablution facilities. Water is either accessed from taps in the yard or from the main house. Backyard dwellers generally make use of the bathroom facilities located inside the main house and these are shared not only with the household living in the formal house but often also amongst multiple backyard households, comprising several families in some instances. Commonly as many as eight people share one bathroom, but in one case over sixteen were reported. This obviously exceeds the number of users that the bathrooms were originally designed to accommodate, compromising hygiene. The literature suggests that constrained access to such services drives up health risks, creating unsanitary conditions and the possibility of contamination and inopportune infections (Dannenberg *et al*. 2003; Govender, Barnes & Piper 2011).

Vredendal backyarders reported that access to the main house is generally not possible at night and when the landlord or landlady is absent, at which time the house is locked. This prevents access to ablution facilities, forcing many backyarders to resort to using the yard, disposing of grey water and human waste indiscriminately, particularly when there are no drains located in the yard for this purpose. This has consequences for the health security, not only of the backyard dwellers but also of members of the host household, reiterating the findings of a study by Govender *et al*. (2011).

Vredendal backyarders expressed a very real fear of the threat of fire, ranking it as a high priority risk during the workshop. Living in generally crowded conditions, with limited room for the storage of personal effects that include bedding materials, cooking and heating appliances and other electrical devices, backyard dwellers live with the constant threat of fire. Concealed behind garden fences or walls, generally out of sight of the street, backyard fires often go unnoticed until they have reached an advanced stage, whilst the nearest emergency services are currently located several kilometres away on the other side of the Olifantsriver in the town of Vredendal, resulting in slow response times. Unlike informal settlements where community members will generally fight fire together to defend common interests, backyard dwellers lack similar social capital (Lemanski 2009).

Informal electricity connections also pose a high fire danger, generally involving an extension cord run from the main house, to which is connected the ubiquitous ‘multiplug’. This provides power to a range of appliances, typically a television, often a radio and even a fridge. Most backyarders are dependent on the landlord for access to electrical power, whilst only a few have metered electricity boxes of their own. Tenants are commonly charged inflated rates for electricity supply and often reduce usage by employing alternative fire-prone cooking, lighting and heating methods such as paraffin stoves and candles. According to the municipality the supply of electricity in Vredendal, which is already constrained, is being exacerbated, both by natural population growth but also by the continued influx of people that is driving up energy demand. Obliged to provide basic electricity supply to the growing informal settlement areas on the periphery of Vredendal North, the additional unmonitored usage by backyarders in the formal housing areas is challenging local capacity whilst also undermining economic expansion.

Lemanski (2009) acknowledges that backyard dwellers are ‘ultimately sharing infrastructure designed for a single household’ and stresses the need for infrastructural upgrading and extended planning to meet the growing demand for basic services amongst backyard dwellers. However, the reality is that small towns with less significant revenue bases have reduced capacity to meet this need.

## Small town versus metropolitan

### Exploring parallels and differences

The research revealed the many inherent similarities to be found amongst backyard dwelling, no matter their geographical context. However, the research also revealed some fundamental differences between city-based backyard dwellers and those living in Vredendal North. These are now explored.

Vredendal North has a particular development history that has resulted in the proliferation of backyard dwellings. Originally a close-knit mixed-race community once located closer to the town of Vredendal in an area called Eureka, residents were forcibly removed to a peripheral location later called Vredendal North in accordance with separate development policies. Squatter communities from several outlying areas were later also relocated there, so that housing demand soon outstripped supply. Originally built as municipally-owned rental accommodation, the several hundred state-built houses were gradually sold off to tenants during the 1980s, whilst several state-subsidy programmes have contributed more houses in recent years. However, supply continues to exceed demand as the large waiting list and spread of backyard dwellings demonstrate. With the influx of migrant work-seekers, several informal settlements have developed on the periphery of Vredendal North, generally settled by black South Africans and foreign migrants. There is much resentment by Vredendal North established residents towards the settlement dwellers, who are considered outsiders competing for limited resources, particularly housing and job opportunities. The legacy of forced removals partially explains why local backyard accommodation in Vredendal North today is so fiercely guarded against outsiders and generally reserved for locals and kin from outlying areas, contrasting with Gunter’s 2014 study in Alexandra, Johannesburg where many foreign migrants rent backyard accommodation from locals. However, Morange (2002) also found a definite racial bias in Port Elizabeth that is perpetuating past housing geographies.

Backyards in metropolitan areas of South Africa are generally considered flexible short term housing options allowing for shifts in employment and access to services, a transitional stage before the provision of formal housing (Morange 2002; Shapurjee & Charlton 2013). In the study area, however, residential mobility is limited and tenancy seldom changes, with most work opportunities generally located in or near Vredendal. In other words, for local residents, backyard rental accommodation is generally the only available housing option, with tenancy only shifting in response to changing household economic circumstances.

However, a key causal factor underlying the poor living conditions and endemic poverty in Vredendal North was also revealed during the fieldwork, not only amongst the backyard dwellers but also amongst the community generally – this is the legacy of the ‘*dop*’ system (London 2000; Scully 1992). Historically in the Western Cape, farm labourers were paid a portion of their wages in a wine ration (Scully 1992). Young children and even babies were fed wine from an early age, a tradition perpetuating alcohol dependency and foetal alcohol syndrome even today. Many farm workers who moved to towns after democratic change in 1994 had to adapt to an unfamiliar urban, more commodified and cash-based existence, with many resorting to the manufacture of traditional home-made wine to supplement household income with an easy and willing local market.

High absenteeism due to heavy drinking partly drove the mechanisation of agriculture and the preference of farmers for foreign labourers (local farmer pers. comm., 2012), whilst the provision of state welfare grants has encouraged grant dependency over poorly paid menial labour (workshop comments, 2012). Local shops in Vredendal North have adapted by extending credit to grant holders, holding grant cards as collateral to ensure repayment – perpetuating the cycle of poverty. According to workshop participants the more recent arrival of home-manufactured drugs like ‘*tik*’ (crystal-amphetamine) has had an easy up-take in this historically substance-dependent culture, influencing school drop-out rates and driving up high levels of crime to support drug habits.

Thus, whilst basic living conditions and everyday hazards amongst the Vredendal North backyarders were found to be similar to those identified in other South African metropolitan studies, notably in Port Elizabeth (Morange 2002), Johannesburg (Gunter 2014) and Cape Town (Lemanski 2009), the study revealed some fundamental differences influencing, not only the choice of backyard accommodation, but also specific local risk drivers. These factors may to some extent determine a certain accepted level of vulnerability amongst local backyard dwellers.

## Conclusion and recommendations

Backyard dwellings, whilst answering to the accommodation needs of a growing number of urban dwellers, can also heighten their exposure to hazards, posing challenges, not only in large urban areas, but increasingly, as this research has illustrated, also in small towns (Rogerson, Kotze & Rogerson 2014). According to Moser (1998), household vulnerability needs to be understood both in terms of longer-term trends as well as more immediate shocks. This research reiterates the importance of understanding both these temporal scales, revealing how particular local histories and changing social dynamics over time have shaped the way in which informal processes function, livelihoods are structured and vulnerabilities determined at the local level, suggesting the need for similar small town studies and the importance of community-led research.

The prevalence of backyard accommodation in Vredendal is likely to increase into the future in light of the shortage of housing, population growth as well as fundamental developmental and social problems. Thus, if we are to accept the inevitability of growing numbers of backyard dwellers in the future, effective and appropriate future planning must incorporate solutions informed, not only by municipal planners and outside experts, but more importantly by those who live these day to day urban realities.

## Footnotes

1 A ‘Wendy house’ refers to a small wooden house usually purchased for use as a children’s playhouse or garden shed. In South Africa, it is frequently used to accommodate backyard dwellers.
